# Comparison of the incidence of slow flow following rotational atherectomy to severely calcified coronary artery lesions between short single session and long single session strategies: the randomized ROTASOLO trial

**DOI:** 10.1007/s12928-025-01177-8

**Published:** 2025-07-23

**Authors:** Kenichi Sakakura, Hiroyuki Jinnouchi, Yousuke Taniguchi, Kei Yamamoto, Yoshimasa Tsurumaki, Takunori Tsukui, Yusuke Watanabe, Takaaki Mase, Masaru Seguchi, Taku Kasahara, Masashi Hatori, Shun Ishibashi, Hiroshi Wada, Yusuke Tamanaha, Kenshiro Arao, Norifumi Kubo, Hideo Fujita

**Affiliations:** 1 Division of Cardiovascular Medicine, Saitama Medical Center, Jichi Medical University, 1-847 Amanuma, Omiya, Saitama City, 330-8503 Japan; 2 Department of Cardiology, JCHO Saitama Medical Center, Saitama City, Japan; 3 Division of Cardiovascular Medicine, Nerima-Hikarigaoka Hospital, Nerima, Japan

**Keywords:** Rotational atherectomy, Percutaneous coronary intervention, Slow flow, Complications, Randomized control study

## Abstract

Slow flow is the most common complication of rotational atherectomy (RA). Compared with long single sessions, short single sessions may reduce the incidence of slow flow just after RA. This study aimed to compare the incidence of slow flow just after RA between short single session and long single session strategies. This multicenter, 1:1 randomized clinical trial was conducted at 3 hospitals in Japan. The short single session strategy was defined as repeating short single sessions (up to 15 s) of RA, whereas the long single session strategy was defined as repeating long single sessions (20–30 s) until the burr crossed the target lesion. The primary outcome was slow flow just after RA, which was defined as [(initial TIMI-frame count before RA) × 1.1 minus (TIMI-frame count just after RA)] less than 0. During the study period, 266 patients were included in the final study population and were randomly assigned to the Short single session group (*n* = 132) or the long single session group (*n* = 134). The protocol adherence rate was equally high in both groups (Short single session: 98.5% versus long single session 94.8%, *p* = 0.172). The incidence of slow flow just after RA was similar between the 2 groups (short single session:14.4% versus long single session: 14.9%, *p* > 0.999). In conclusion, this randomized trial did not show a benefit of the short single session strategy compared with the long single session strategy in RA with respect to the prevention of slow flow (Unique identifier: UMIN000047231).

**Graphical abstract:**

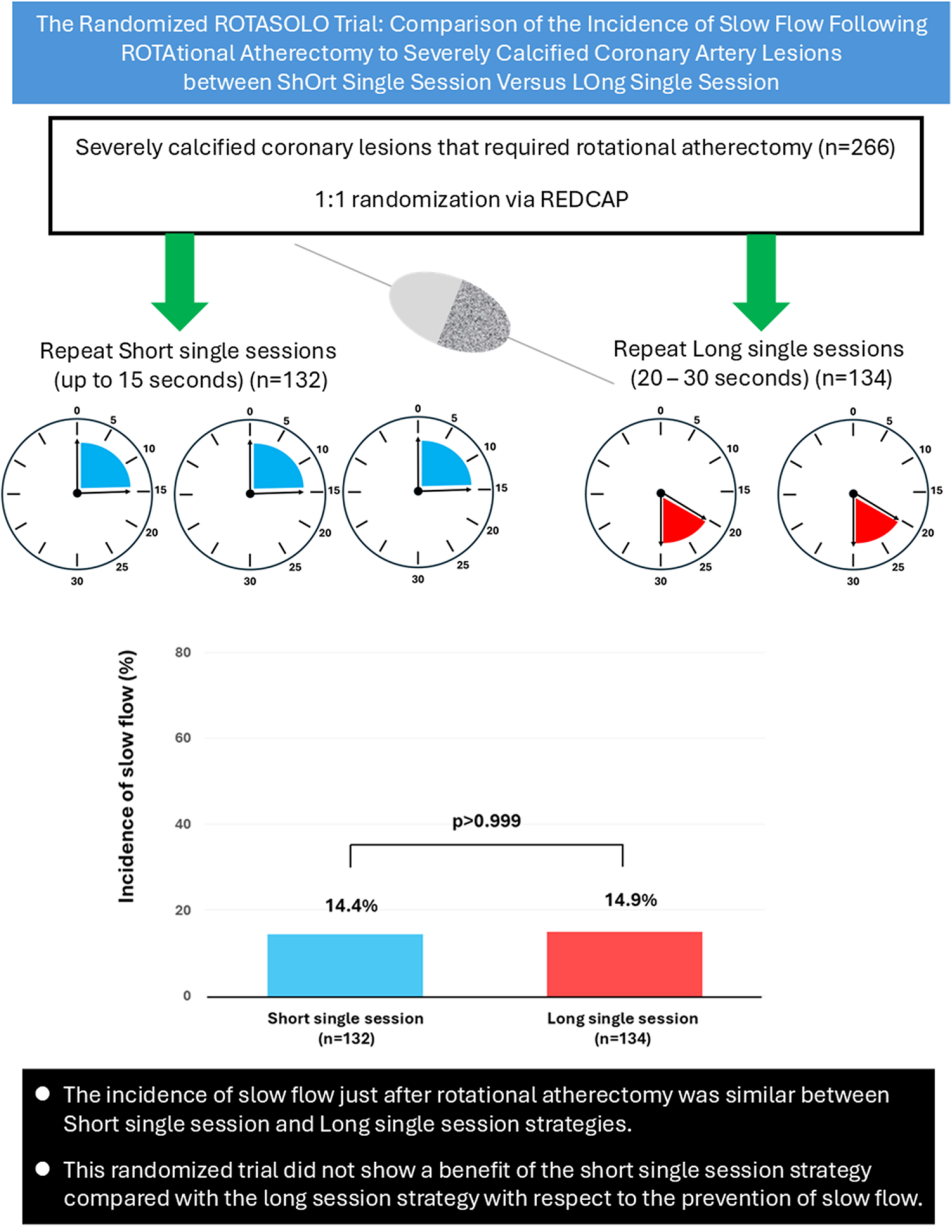

## Introduction

Severe calcification is closely associated with poor outcomes in the contemporary percutaneous coronary intervention (PCI) [1, 2]. Rotational atherectomy (RA) has been used for severely calcified coronary artery disease (CAD) for more than 2 decades [3–5]. Although new procedures, including orbital atherectomy and intravascular lithotripsy, have emerged for severely calcified CAD, RA still plays an important role in the treatment of severely calcified CAD [6–8]. However, specific complications such as burr entrapment occur in PCI with RA [9–12]. Among these, slow flow is the most common [13–15]. The severity of slow flow varies widely from transient thrombolysis in myocardial infarction (TIMI) grade 2 flow to persistent TIMI grade 0 flow (no flow), which results in serious periprocedural myocardial infarction (PMI) [16–18]. Previous retrospective studies found that slow flow following RA was associated with lesion length, angulation, and burr-to-artery ratio, reference diameter, systolic blood pressure just before RA, and primary RA strategy [13, 19]. Most of these characteristics, such as lesion length or angulation are unmodifiable factors in RA. Even total ablation time is not a modifiable factor in RA, because diffuse long lesions naturally require a long total ablation time. However, single run time is a modifiable factor by operators.

Although the clinical expert consensus document from the Japanese Association of CardioVascular Intervention and Therapeutics (CVIT) recommends appropriate burr size, short ablation time (short single session), and avoiding excessive speed down [20], methods to prevent slow flow have not been established and evidence supporting such a recommendation is sparse. Because our retrospective study showed that the short single session (≤ 15 s) strategy was inversely associated with slow flow [13], we hypothesized that this strategy would prevent the occurrence of slow flow following RA irrespective of total ablation time. This paper describes the main results of the trial entitled “Comparison of the Incidence of Slow Flow Following ROTAtional Atherectomy to Severely Calcified Coronary Artery Lesions between ShOrt Single Session Versus LOng Single Session: The Randomized ROTASOLO Trial” [UMIN000047231].

## Methods

### Study design

The design of the ROTASOLO trial, which is a prospective, multi-center, investigator-initiated, randomized study, has been reported previously [21]. In brief, patients who underwent RA were randomly assigned in a 1:1 ratio to the short single session strategy and the long single session strategy. The short single session strategy was defined as repeating short single sessions (up to 15 s) of RA until the burr crossed the target lesion, whereas the long single session strategy was defined as repeating long single sessions (20–30 s) of RA until the burr crossed the target lesion. The study patients were recruited from the following 3 hospitals: (1) Saitama Medical Center, Jichi Medical University, (2) JCHO Saitama Medical Center, and (3) Nerima-Hikarigaoka Hospital in Japan. The enrollment period was 35 months (April 2022 to February 2025). The primary outcome was assessed immediately after RA in each procedure. The study was approved by the Institutional Review Board of each hospital. The date of disclosure of the study information was March 25, 2022 at UMIN-CTR Clinical Trial (https://www.umin.ac.jp/ctr/index.htm: Unique ID: UMIN000047231), which was done before the enrollment period. The enrollment was completed on February 19, 2025 and the trial data were considered complete (data were fixed) on March 12, 2025 after the data monitoring by the Center for Clinical Investigation of Jichi Medical School.

Inclusion criteria for participation in the ROTASOLO trial were as follows: (1) patients with ischemic heart disease including acute coronary syndrome and chronic coronary syndrome who underwent PCI using RA, (2) patients who gave written informed consent, (3) angiographically severe calcification in target lesions, and (4) and intravascular imaging showing an over 180-degree superficial calcification/calcified nodule, intravascular imaging devices unable to cross the lesion due to severe stenosis, or intravascular imaging device (typically optical coherent tomography) unable to provide valid images due to severe stenosis [21]. Exclusion criteria were as follows: (1) patients less than 20 years of age and (2) patients with a contraindication listed in the Rotablator instructions for use [21].

### Randomization

Pre-screening was performed by investigators according to the findings of coronary angiography and/or computed tomography (CT) angiography. Investigators made a tentative registration for the study via REDCap (Research Electronic Data Capture; Vanderbilt University) [22, 23]. During PCI, investigators confirmed that patients had angiographically severe calcification and met intravascular imaging criteria. Then, the operators decided to perform RA and chose the initial burr size and the type of RotaWire before randomization. Patients were centrally randomized in a 1:1 ratio using REDCap. Although this study included patients with acute coronary syndrome, including ST-segment elevation myocardial infarction (STEMI), no emergent RA cases were included. RA was deferred in emergent PCI and was scheduled as elective PCI in this study participants, partly because the risk of complications in RA is much greater in emergent PCI than in elective PCI [24].

The ROTAPRO (Boston Scientific, Marlborough, MA) device was used for all RA procedures. The RA burr was advanced over the wire to a position proximal to the lesion. The rotational speed was set at the conventional range (140,000–190,000 rpm) with the burr proximal to the lesion. Techniques regarding RA were consistent with those recommended by the clinical expert consensus document on RA from the Japanese Association of Cardiovascular Intervention and Therapeutics [20, 25]. In the short single-session group, operators controlled the single session time up to 15 s. In the long single session group, operators controlled the single session time from 20 to 30 s. In both groups, operators could add sessions until the first burr crossed the target lesion. There was no restriction regarding the interval time between sessions. Operators could take sufficient time freely between sessions. If operators decided to use the second burr (i.e. burr size-up) after the first burr crossed the target lesion, operators could set the single session time freely. In other words, operators did not need to follow the short or long single session strategy after the first burr crossed the target lesion. The console of the ROTAPRO device clearly displayed each run time, which was visible to the main- and sub-operators.

### Primary outcome

The primary outcome was slow flow just after RA. In the ROTASOLO trial, slow flow just after RA was defined as [(initial TIMI-frame count before RA) × 1.1 minus (TIMI-frame count just after RA)] less than 0 [21]. Absence of slow flow was defined as [(initial TIMI-frame count before RA) × 1.1 minus (TIMI frame count just after RA)] not lower than 0 [21]. For the TIMI-frame count evaluation, we set the frame rate as 15 frames per second (15 fps). We multiplied the initial TIMI-frame count before RA 1.1-fold, because the TIMI frame count would be influenced not only by slow flow, but also by injection speed, dose of the contrast media, depth of the guide-catheter, and presence of the guidewire. In other words, if the TIMI-frame count just after RA was slightly higher than that before RA, it may represent a margin of error rather than slow flow caused by RA. Therefore, we compared the initial TIMI-frame count before RA × 1.1 with the TIMI-frame count just after RA.

If ≥ 2 burrs were used for RA, slow flow was evaluated only after the first burr crossed the lesion. Once the first burr crossed the lesion, slow flow was not evaluated for this study after the second burr crossed the lesion. If the first burr could not cross the lesion and the second burr (typically a smaller burr) could cross the lesion, slow flow was evaluated for this study after the second burr crossed the lesion. If halfway RA was performed [26], slow flow was evaluated just after halfway RA. In other words, slow flow just after RA was evaluated only one time per PCI. The detail of timing when we evaluate slow flow just after RA were described previously [21]. Secondary outcomes were PMI and complications such as vessel perforation.

### Definitions of variables

All clinical information, including patient, lesion, and procedure characteristics, and study outcomes, was collected as electronic data capture (EDC) via REDCap [21]. The definition of hypertension, diabetes mellitus, and dyslipidemia were described previously [21]. Creatine kinase (CK) and creatine kinase-myocardial band (CK-MB) on the day after RA were collected. Serial measurements for CK and CK-MB were not performed. PMI was defined as CK-MB ≥ 10 upper limit of normal [27]. Even in the patients with acute coronary syndrome, we did not perform any adjustment for the definition of PMI. The reference diameter and lesion length were calculated by quantitative coronary angiography [13]. The burr-to-artery ratio was defined as the burr size divided by the reference diameter [13].

### Sample size calculations and statistical methods

Sample size calculations were based on previously published data. In the retrospective study that included 513 lesions treated with RA, the incidence of slow flow was 14.7% in lesions that received short single sessions (no more than 15 s), whereas the incidence of slow flow was 28.8% in lesions that received long single sessions (20–30 s) [13]. For a cut-off of the probability of a type-I error (*α*) of 5% (0.05) and a type-II error (*β*) of 20% (0.2), a total of 266 lesions was needed to detect the difference between the 2 groups. We anticipated that a substantial number of cases would be excluded by our strict imaging criteria; thus, 300 patients from tentative registration and 266 patients from formal registration were chosen as the sample size for the ROTASOLO study. The ROTASOLO study was monitored and audited by the Center for Clinical Investigation of Jichi Medical School [21].

Data are presented as a percentage for categorical variables or a median and inter-quartile range for non-normally distributed continuous variables. The primary outcome (incidence of slow flow) was compared between the short single session group and the long single session group using Fischer’s exact test. The Wilk-Shapiro test was performed to determine if the continuous variables were normally distributed. Non-normally distributed continuous variables were compared between the two groups using the Mann–Whitney *U* test. Categorical data were compared using Fischer’s exact test or Fischer–Freeman–Halton exact test. In addition, we performed subgroup analyses to investigate the effects of age, sex, hemodialysis, history of heart failure admission, use of beta blockers, chronic coronary syndrome, reference diameter, lesion length, lesion angle, and initial burr-to-artery ratio on the primary outcome. The treatment-by-subgroup interaction was assessed for all subgroups, and the odds between Short single session and Long single session (reference) were calculated for each subgroup. All reported *p* values were determined by two-sided analysis, and *p* values < 0.05 were considered significant. All analyses were performed with IBM SPSS statistics version 29 (Chicago, IL, USA).

## Results

**Fig. 1:**
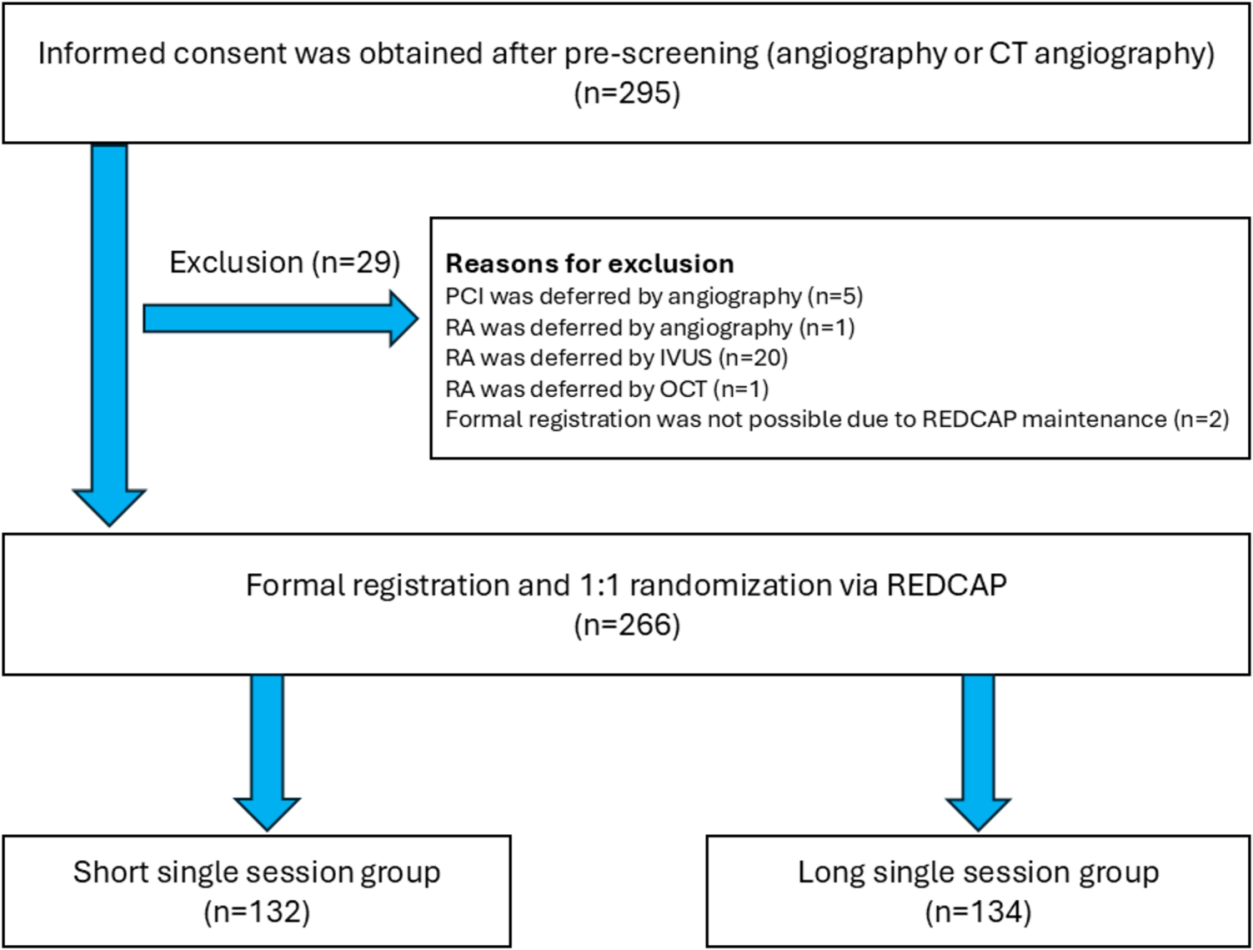
Patient flow diagram. *CT* computed tomography, *PCI* percutaneous coronary intervention, *RA* rotational atherectomy, *IVUS* intravascular ultrasound, *OCT* optical coherence tomography

During the study period (April 2022 to February 2025), 3569 PCIs were conducted in 3 participating hospitals. Among those, 355 RA were performed (RA rate, 9.9%). After exclusion of 29 patients, 266 patients were included in the final study population and were randomly assigned to the Short single session group (*n* = 132) and the Long single session group (*n* = 134). The patient flow diagram is shown in Fig. 1.

**Fig. 2:**
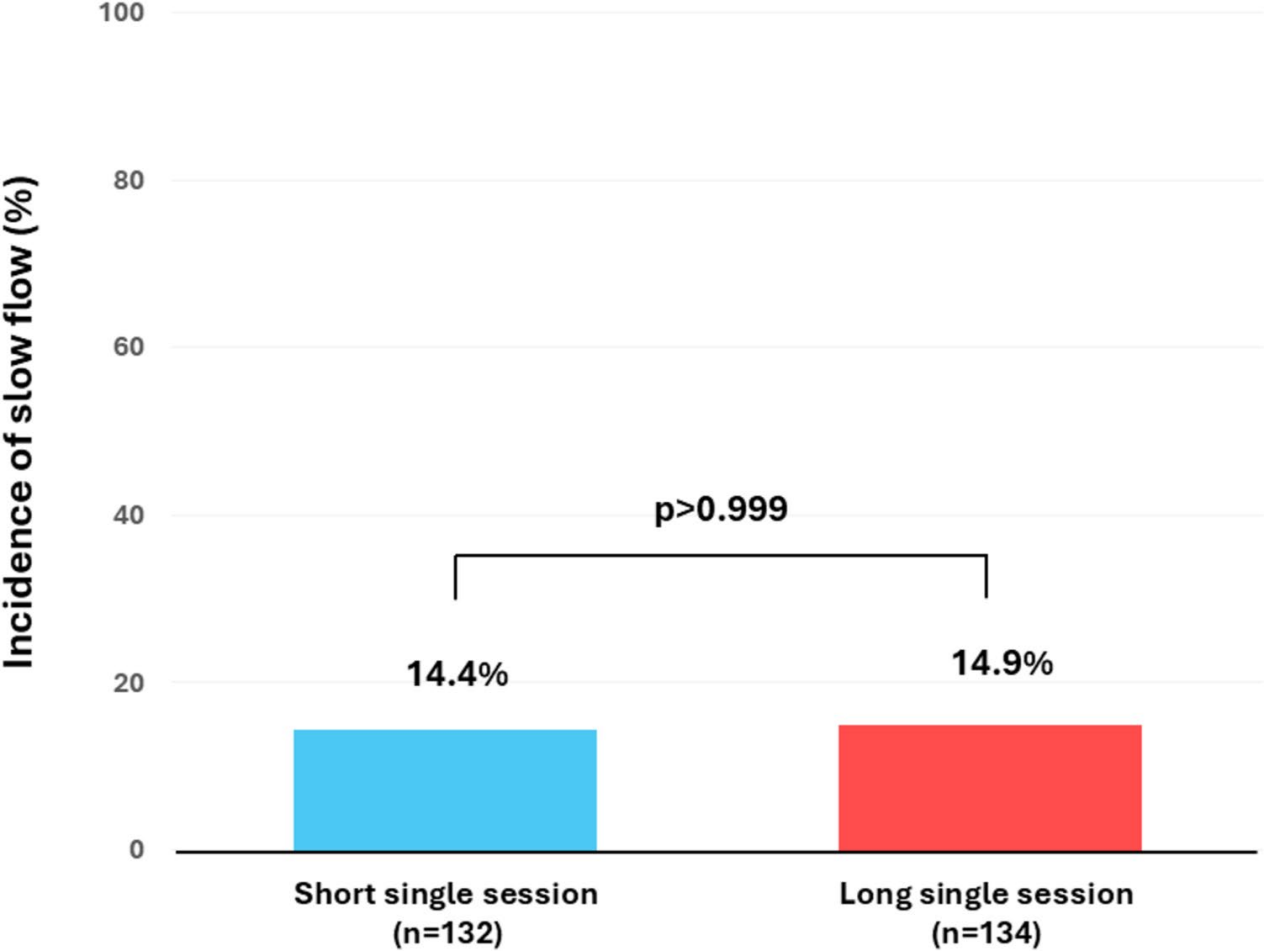
Incidence of slow flow just after rotational atherectomy between the short single session and long single session groups. Fischer’s exact test

Patient and lesion characteristics for the 2 groups are shown in Table 1. There were no significant differences between the 2 groups. The incidence of slow flow just after RA (primary outcome) is shown in Fig. 2. The incidence of slow flow was similar between the 2 groups (Short single session:14.4% versus Long single session: 14.9%, *p* > 0.999). Procedure characteristics for the 2 groups are shown in Table 2. The number of sessions until primary outcome evaluation was significantly higher in the Short single session group than in the Long single session group, whereas total RA time until primary outcome evaluation was not significantly different between the 2 groups. The protocol adherence rate was equally high in both groups. Other procedural variables, including the number of burr size, types of RotaWire, burr-to-artery ratio, and final procedure were comparable. All cases achieved a final TIMI flow grade 3.

**Table 1:** Patient and lesion characteristics between the short single session and long single session groups

Variable	All (*n* = 266)	Short single session (up to 15 s) (*n* = 132)	Long single session (20–30 s) (*n* = 134)	*p* value
Patient characteristics				
Age (years)	77.0 (70.0–81.0)	77.0 (70.0–81.0)	76.0 (69.0–81.0)	0.547
Male sex—no. (%)	204 (76.7)	106 (80.3)	98 (73.1)	0.193
Body mass index (kg/m^2^)	22.9 (20.8–25.7)	22.7 (20.8–25.6)	23.1 (20.8–25.8)	0.947
Hypertension—no. (%)	256 (96.2)	127 (96.2)	129 (96.3)	> 0.999
Diabetes mellitus—no. (%)	165 (62.0)	80 (60.6)	85 (63.4)	0.705
Dyslipidemia—no. (%)	259 (97.4)	129 (97.7)	130 (97.0)	> 0.999
Current smoker—no. (%)	40 (15.0)	18 (13.6)	22 (16.4)	0.608
Hemodialysis—no. (%)	83 (31.2)	38 (28.8)	45 (33.6)	0.429
Peritoneal dialysis—no. (%)	6 (2.3)	3 (2.3)	2 (2.2)	> 0.999
History of heart failure admission—no. (%)	48 (18.0)	25 (18.9)	23 (17.2)	0.751
Medications				
Statin—no. (%)	253 (95.1)	126 (95.5)	127 (94.8)	> 0.999
ACEi/ARB/ARNI—no. (%)	166 (62.4)	86 (65.2)	80 (59.7)	0.378
Beta blockers—no. (%)	199 (74.8)	96 (72.7)	103 (76.9)	0.481
Creatinine at admission (mg/dL)	1.08 (0.77–6.35)	1.08 (0.77–5.53)	1.08 (0.80–7.11)	0.694
Ejection fraction (%)	59.4 (44.2–64.3) (*n* = 230)	59.1 (43.3–64.2) (*n* = 113)	59.9 (45.0–65.0) (*n* = 117)	0.721
Lesion characteristics				
Clinical presentation				0.483
STEMI—no. (%)	9 (3.4)	3 (2.3)	6 (4.5)	
NSTEMI—no. (%)	29 (10.9)	13 (9.8)	16 (11.9)	
Unstable angina—no. (%)	9 (3.4)	3 (2.3)	6 (4.5)	
Chronic coronary syndrome—no. (%)	219 (82.3)	113 (85.6)	106 (79.1)	
Angiographically severe calcification	266 (100)	132 (100)	134 (100)	-
Visible thrombus—no. (%)	0	0	0	-
RA for chronic total occlusion—no. (%)	1 (0.4)	0	1 (0.7)	> 0.999
In-stent lesion—no. (%)	7 (2.6)	5 (3.8)	2 (1.5)	0.280
Target lesion				0.367
Left main-LAD—no. (%)	187 (70.3)	90 (68.2)	97 (72.4)	
LCX—no. (%)	20 (7.5)	13 (9.8)	7 (5.2)	
RCA—no. (%)	59 (22.2)	29 (22.0)	30 (22.4)	
Left main ostium—no. (%)	3 (1.1)	1 (0.8)	2 (1.5)	> 0.999
LAD ostium—no. (%)	31 (11.7)	14 (10.6)	17 (12.7)	0.703
LCX ostium—no. (%)	12 (4.5)	9 (6.8)	3 (2.2)	0.083
RCA ostium—no. (%)	14 (5.3)	7 (5.3)	7 (5.2)	> 0.999
Reference diameter (mm)	2.50 (2.19–2.80)	2.40 (3.10–2.90)	2.50 (2.19–2.80)	0.921
Lesion length (mm)	22.05 (11.00–35.00)	21.35 (10.54–33.35)	22.55 (11.70–35.40)	0.465
Lesion angle				0.061
Mild (< 30 degrees)—no. (%)	156 (58.6)	73 (55.3)	83 (61.9)	
Moderate (30–60 degrees)—no. (%)	90 (33.8)	44 (33.3)	46 (34.3)	
Severe (≥ 60 degrees)—no. (%)	20 (7.5)	15 (11.4)	5 (3.7)	
Intravascular imaging before RA (imaging criteria)				0.197
IVUS was tried, but IVUS catheter could not cross the lesion—no. (%)	101 (38.0)	57 (43.2)	44 (32.8)	
IVUS revealed over 180-degree superficial calcification/calcified nodule—no. (%)	148 (55.6)	69 (52.3)	79 (59.0)	
OCT was tried, but OCT catheter could not cross the lesion—no. (%)	6 (2.3)	2 (1.5)	4 (3.0)	
OCT was tried, but adequate image was not available due to severe stenosis—no. (%)	1 (0.4)	1 (0.8)	0 (0)	
OCT revealed over 180-degree superficial calcification/calcified nodule—no. (%)	10 (3.8)	3 (2.3)	7 (5.2)	
Degree of superficial calcification/calcified nodule				0.061
180–270—no. (%)	53 (19.9)	24 (18.2)	29 (21.6)	
270–360—no. (%)	49 (18.4)	28 (21.2)	21 (15.7)	
360—no. (%)	56 (21.1)	20 (15.2)	36 (26.9)	
Imaging catheter uncrossed or adequate image was not available—no. (%)	108 (40.6)	60 (45.5)	48 (35.8)	
Balloon dilatation before imaging study—no. (%)	19 (7.1)	12 (9.1)	7 (5.2)	0.243

Data are expressed as median (Q1–Q3) or number (percentage). The Mann–Whitney *U* test was used for continuous variables, and the Fisher's exact test or Fischer–Freeman–Halton exact test was used for categorical variables

*ACEi* angiotensin converting enzyme inhibitors, *ARB* angiotensin receptor blockers, *ARNI* angiotensin receptor neprilysin inhibitor, *STEMI ST*-segment elevation myocardial infarction, *NSTEMI* non-ST-segment elevation myocardial infarction, *RA* rotational atherectomy, *LAD* left anterior descending artery, *LCX* left circumflex artery, *RCA* right coronary artery, *IVUS* intravascular ultrasound, *OCT* optical coherence tomography

**Table 2:** Procedure characteristics between the short single session and long single session groups

Variable	All (*n* = 266)	Short single session (up to 15 s) (*n* = 132)	Long single session (20–30 s) (*n* = 134)	*p* value
TIMI flow grade before RA				0.355
TIMI 1—*n* (%)	2 (0.8)	2 (1.5)	0 (0)	
TIMI 2—*n* (%)	28 (10.5)	12 (9.1)	16 (11.9)	
TIMI 3—*n* (%)	236 (88.7)	118 (89.4)	118 (88.1)	
TIMI frame count before RA	18.0 (14.0–24.0)	19.0 (13.5–24.0)	18.0 (14.0–24.0)	0.801
TIMI flow grade just after RA (primary outcome evaluation)				0.838
TIMI 1—*n* (%)	3 (1.1)	2 (1.5)	1 (0.7)	
TIMI 2—*n* (%)	14 (5.3)	6 (4.5)	8 (6.0)	
TIMI 3—*n* (%)	249 (93.6)	124 (93.9)	125 (93.3)	
TIMI frame count just after RA (primary outcome evaluation)	14.0 (11.0–20.0)	15.0 (12.0–20.0)	14.0 (11.0–20.0)	0.360
Used burr size for primary outcome evaluation				0.835
1.25 mm—*n* (%)	20 (7.5)	12 (9.1)	8 (6.0)	
1.5 mm—*n* (%)	219 (82.3)	108 (81.8)	111 (82.8)	
1.75 mm—*n* (%)	22 (8.3)	10 (7.6)	12 (9.0)	
2.0 mm—*n* (%)	4 (1.5)	2 (1.5)	2 (1.5)	
2.15 mm—n (%)	1 (0.4)	0 (0)	1 (0.7)	
Number of sessions until primary outcome evaluation	3.0 (2.0–4.0)	4.0 (3.0–6.0)	2.0 (1.0–3.0)	< 0.001
Mean single session time until primary outcome evaluation (seconds)	20.0 (10.0–22.0)	10.0 (9.0–11.2)	22.0 (21.3–22.5)	< 0.001
Total RA time until primary outcome evaluation (seconds)	42.0 (24.0–63.9)	36.0 (25.6–59.5)	44.0 (23.0–66.0)	0.319
Protocol adherence—*n* (%)	257 (96.6)	130 (98.5)	127 (94.8)	0.172
Balloon dilatation before RA—*n* (%)	20 (7.5)	12 (9.1)	8 (6.0)	0.362
Guide catheter size				0.293
6-Fr—*n* (%)	21 (7.9)	14 (10.6)	7 (5.2)	
7-Fr—*n* (%)	226 (85.0)	109 (82.6)	117 (87.3)	
8-Fr—*n* (%)	19 (7.1)	9 (6.8)	10 (7.5)	
Use of intra-aortic balloon pumping—*n* (%)	7 (2.6)	4 (3.0)	3 (2.2)	0.721
Types of RotaWire				0.741
RotaWire drive floppy—*n* (%)	195 (73.3)	99 (75.0)	96 (71.6)	
RotaWire drive extra-support—*n* (%)	68 (25.6)	31 (23.5)	37 (27.6)	
Switch from floppy to extra-support—*n* (%)	2 (0.8)	1 (0.8)	1 (0.7)	
Switch from extra-support to floppy—*n* (%)	1 (0.4)	1 (0.8)	0 (0)	
Number of used burrs during whole procedure				0.949
1	163 (61.3)	81 (61.4)	82 (61.2)	
2	102 (38.3)	50 (37.9)	52 (38.8)	
3	1 (0.4)	1 (0.8)	0 (0)	
Initial burr size				0.975
1.25 mm—*n* (%)	4 (1.5)	2 (1.5)	2 (1.5)	
1.5 mm—*n* (%)	235 (88.3)	118 (89.4)	117 (87.3)	
1.75 mm—*n* (%)	22 (8.3)	10 (7.6)	12 (9.0)	
2.0 mm—*n* (%)	4 (1.5)	2 (1.5)	2 (1.5)	
2.15 mm—*n* (%)	1 (0.4)	0 (0)	1 (0.7)	
Maximum burr size				0.863
1.25 mm—*n* (%)	2 (0.8)	1 (0.8)	1 (0.7)	
1.5 mm—*n* (%)	163 (61.3)	83 (62.9)	80 (59.7)	
1.75 mm—*n* (%)	51 (19.2)	26 (19.7)	25 (18.7)	
2.0 mm—*n* (%)	43 (16.2)	20 (15.2)	23 (17.2)	
2.15 mm—*n* (%)	5 (1.9)	1 (0.8)	4 (3.0)	
2.25 mm—*n* (%)	2 (0.8)	1 (0.8)	1 (0.7)	
Burr-to-artery ratio (initial burr size)	0.63 (0.54–0.71)	0.63 (0.54–0.71)	0.61 (0.54–0.71)	0.917
Burr-to-artery ratio (maximum burr size)	0.67 (0.58–0.77)	0.65 (0.56–0.78)	0.68 (0.59–0.77)	0.689
Total RA time (whole RA procedure) (second)	45.0 (28.0–76.0)	43.0 (27.5–71.5)	45.0 (28.0–76.0)	0.287
Mean single session time (whole RA procedure) (second)	12.8 (10.0–21.0)	9.9 (8.7–11.2)	21.0 (19.0–22.0)	< 0.001
Mean rotational speed (rpm)	183,619 (180,500–187,000)	183,212 (180,572–186,666)	184,000 (180,500–188,000)	0.192
Systolic blood pressure just before RA (mmHg)	146.0 (130.0–159.0)	144.5 (130.0–158.0)	147.0 (132.0–160.0)	0.571
Diastolic blood pressure just before RA (mmHg)	72.0 (66.0–80.0)	71.0 (65.0–79.0)	74.0 (67.0–82.0)	0.051
Heart rate just before RA (bpm)	70.0 (63.0–78.0)	69.5 (61.0–77.0)	72.0 (65.0–80.0)	0.055
Halfway rotational atherectomy—*n* (%)	15 (5.6)	8 (6.1)	7 (5.2)	0.797
Final procedure				0.447
RA and drug-eluting stent—*n* (%)	125 (47.0)	57 (43.2)	68 (50.7)	
RA and ballooning including drug-coated balloon—*n* (%)	121 (45.5)	65 (49.2)	56 (41.8)	
Others^a^—*n* (%)	20 (7.5)	10 (7.6)	10 (7.5)	
Final TIMI flow grade				-
TIMI ≤ 2—*n* (%)	0	0	0	
TIMI 3—*n* (%)	266 (100)	132 (100)	134 (100)	

Data are expressed as median (Q1–Q3) or number (percentage). The Mann–Whitney *U* test was used for continuous variables, and the Fisher's exact test or Fischer–Freeman–Halton exact test was used for categorical variables

*TIMI* thrombolysis in myocardial infarction

^a^Others include 1 case of RA alone and 19 cases of the combination of RA, drug-eluting stent, and drug-coated balloon

Complications between the 2 groups are shown in Table 3. There was no type III perforation in either group. One burr entrapment occurred in the short single session group, which was successfully resolved by simply pulling the system. One in-hospital death due to cholecystitis occurred in the short single session group. CK and CK-MB were comparable between the 2 groups. There were no cases of periprocedural myocardial infarction.

**Table 3:** Complications between the short single session and long single session groups

Variable	All (*n* = 266)	Short single session (up to 15 s) (*n* = 132)	Long single session (20–30 s) (*n* = 134)	*p* value
Type III perforation due to RA burr	0	0	0	–
Type III perforation due to guidewire	0	0	0	–
Type III perforation due to ballooning or stenting	0	0	0	–
Burr entrapment	1 (0.4)	1 (0.8)	0	0.496
In-hospital death	1 (0.4)	1 (0.8)	0	0.496
Creatine kinase at next morning	89.0 (59.0–135.0) (*n* = 265)	89.0 (57.5–130.5) (*n* = 131)	89.5 (62.0–141.0)	0.459
Creatine kinase-myocardial band at next morning	5.0 (3.0–9.0) (*n* = 265)	4.0 (3.0–8.5) (*n* = 131)	5.0 (3.0–10.0)	0.101
Periprocedural myocardial infarction	0 (*n* = 265)	0 (*n* = 131)	0	–

Data are expressed as median (Q1–Q3) or number (percentage). The Mann–Whitney *U* test was used for continuous variables, and the Fisher’s exact test was used for categorical variables

The results of selected subgroup analysis to compare slow flow between short single session and long single session are shown in Table 4. There was no significant treatment-by-subgroup interaction for the subgroups except for history of heart failure admission. The incidence of slow flow was not different in any of the subgroups between short single session and long single session.

**Table 4:** Subgroup analysis to compare the incidence of slow flow between the short single session and long single session groups

Subgroup	Short single session (up to 15 s) (*n* = 132)	Long single session (20–30 s) (*n* = 134)	Odds ratio	95% confidence interval	*p* value	*p* value for interaction
All cases	132	134	0.958	0.486–1.891	0.902	
Age						0.147
Age < 75 (years)	47	59	1.723	0.620–4.785	0.297	
Age ≥ 75 (years)	85	75	0.622	0.246–1.570	0.315	
Sex						0.435
Male	106	98	1.181	0.523–2.667	0.688	
Female	26	36	0.636	0.169–2.391	0.503	
Hemodialysis						0.773
Yes	38	45	0.823	0.238–2.839	0.757	
No	94	89	1.023	0.452–2.317	0.956	
History of heart failure admission						0.039
Yes	25	23	0.199	0.036–1.084	0.062	
No	107	111	1.424	0.655–3.096	0.373	
Beta blockers use						0.874
Yes	96	103	0.919	0.412–2.047	0.836	
No	36	31	1.04	0.284–3.808	0.953	
Chronic coronary syndrome						0.482
Yes	113	106	1.074	0.507–2.277	0.852	
No	19	28	0.541	0.094–3.132	0.493	
Reference diameter						0.419
< 2.5 mm	70	62	1.186	0.479–2.935	0.712	
≥ 2.5 mm	62	72	0.664	0.227–1.946	0.456	
Lesion length						0.115
< 20 mm	64	59	0.471	0.148–1.496	0.202	
≥ 20 mm	68	75	1.508	0.633–3.596	0.354	
Lesion angle						0.458
Mild	73	83	0.757	0.303–1.891	0.551	
Moderate to severe	59	51	1.283	0.450–3.659	0.641	
Initial burr to artery ratio						0.466
< 0.6	55	53	0.689	0.222–2.139	0.519	
≥ 0.6	77	81	1.168	0.497–2.747	0.722	

Univariate logistic regression analysis was performed in each subgroup. Short single session (versus long single session) was included as independent variable whereas slow flow was included as dependent variable

## Discussion

The results of this randomized trial did not show a benefit of the short single session strategy in RA with respect to the prevention of slow flow. Although major expert consensus documents on RA have been published from Europe, North America, and Japan [20, 28, 29], recommendations to prevent slow flow are not sufficiently supported by robust evidence. Our findings do not match the published data regarding slow flow in retrospective studies and the description in the Japanese expert consensus document, which partly recommends short single sessions. This trial underscores the importance of conducting a randomized trial for the evaluation of RA procedures.

Because the number of RA cases per operator was inversely associated with adverse events [30, 31], refinement of RA procedures is important to reduce complications related to RA. Although several randomized studies assessed the combination of RA and modified balloon or the comparison between RA and modified balloons [32, 33], few have investigated RA procedures or RA techniques. Our group previously conducted a randomized study to compare the incidence of slow flow between low-speed (140,000 rpm: *n* = 50) with high-speed (190,000 rpm: *n* = 50) and revealed that the incidence of slow flow was similar between low-speed and high-speed [34]. Acar, et al. conducted a randomized study to compare the incidence of bradycardia or high-grade atrioventricular block between rota-flush with (*n* = 30) and without aminophylline (*n* = 30), and reported that rota-flush with aminophylline was inversely associated with bradycardia or high-grade atrioventricular block [35]. However, these studies were of relatively small sample size and the definition of slow flow was TIMI flow grade ≤ 2, which might be a subjective indicator. Furthermore, TIMI flow grade ≤ 2 as the definition of slow flow has another problem. If TIMI flow grade 1 before RA improved to TIMI flow grade 2 after RA, it is natural to interpret that RA improved coronary flow. However, this case would be classified as slow flow by its definition. The strengths of the ROTASOLO trial were (1) to adopt a unique definition of slow flow just after RA, (2) to set an adequate sample size for the primary outcome according to the sample size calculation, and (3) to adopt strict intravascular imaging criteria for study inclusion, which were not used in previous studies.

There are several possible explanations for the similar incidences of slow flow between short and long single sessions. In the sample size calculation, we anticipated the incidence of slow flow just after RA in the Short session and Long session groups was 14.7% and 28.8%, respectively. The actual incidence of slow flow in the Short single session group (14.4%) was very similar to the anticipated incidence, whereas the actual incidence of slow flow in the Long session group (14.9%) was much smaller than the anticipated incidence. However, we did the sample size calculation using TIMI flow grade ≤ 2 as the definition of slow flow. In the present study, the incidence of TIMI flow grade ≤ 2 in the Short session and Long session groups was only 6.1% and 6.7%, respectively. In other words, the incidence of TIMI flow grade ≤ 2 just after RA was much smaller than anticipated in both groups. We should note that the study for the sample size calculation included patients from November 2014 to August 2020 in Saitama Medical Center, Jichi Medical University [13], which is the primary center for the present study. On the other hand, the present study included patients from April 2022 to February 2025. There is a time gap between our previous retrospective study and the present study. During the time gap, operators engaged in the present study might increase the understanding of slow flow and develop their skills in the prevention of slow flow during RA, because operators in the present study recently developed several techniques to prevent complications, including slow flow [26, 36]. Moreover, the operators might have made their best effort to minimize the incidence of slow flow especially in the Long session group, because operators were not blinded to the study hypothesis that short single session might reduce the incidence of slow flow. Furthermore, since there was no restriction regarding the time interval between sessions, operators could take sufficient time between sessions when ST- segment elevation was observed. Because ST-segment elevation often precedes slow flow in RA, slow flow might have been prevented by the incorporation of appropriate actions for ST-segment elevation including sufficient interval, intravenous noradrenaline, intracoronary vasodilators, burr size down, and intentional halftime [25, 36].

The clinical implications of this trial should be noted. Since the results do not support short single sessions, operators may not need to shorten the single session time during RA. Operators may extend the single session time up to 20–30 s if needed. The technical issues of RA are roughly divided into 2 key questions: (1) how operators manipulate the burr (quick pecking motion or slow pecking motion?), and (2) how long operators can permit for the single session time (10 s, 15 s, 20 s, or 30 s?). If the single session time is not so important, operators can concentrate on the burr manipulation, which would make RA procedures simpler and easier, especially for junior RA operators. Among 10 subgroup analyses, there was a significant interaction in the history of heart failure admission. In patients with a history of heart failure admission (*n* = 48), the incidence of slow flow was lower in the Short session group than in the long session group without reaching statistical significance (odds ratio 0.199, 95% confidence interval 0.036–1.084, *p* = 0.062). This finding suggests the possibility that the short single session strategy may have differential effects across clinical subgroups and may be beneficial in patients with a history of heart failure admission. Furthermore, several factors such as lesion length, burr-to-artery ratio, and use of beta-blockers are reported to be associated with slow flow after RA in previous studies [13, 19]. However, these previous studies are retrospective study using the subjective definition of slow flow (TIMI flow grade ≤ 2). In the present randomized study, we used a unique definition of slow flow, which may be useful to verify the association between clinical factors and slow flow after RA. Future prospective studies with objective definitions of slow flow are warranted to identify the true causes of slow flow after RA.

The ROTASOLO study had several limitations. First, quantitative coronary angiography and slow flow evaluations were not performed by independent core laboratories. Second, our definition of slow flow using TIMI-frame count has not been used by other groups. Although we did define it before study enrollment [21], its clinical relevance remains unvalidated. Third, the inability to blind operators might have impacted the results. Fourth, our definition of PMI, which used CK-MB as a biomarker, was not sensitive enough to detect minor PMI. Fifth, there were no restrictions or protocols regarding the use of vasodilators before, during, and after RA. However, all three hospitals coincidentally used the same drug cocktail for RA, which was composed of nicorandil 12 mg, isosorbide dinitrate 2.5 mg, heparin 10,000 units, and normal saline 500 mL [15]. Therefore, at least some vasodilators were used during RA. Sixth, some parameters, such as antithrombotic agents, activated coagulation time, and attenuation plaques, which may be potential confounding factors for the incidence of slow flow, were not available in this study. However, randomization is a powerful method to control both known and unknown confounding factors. Finally, investigators might not have considered patients with very severe conditions or those with very complex anatomy as candidates for this randomized trial.

## Conclusions

This randomized trial did not show a benefit of the short single session strategy over the long single session strategy in RA with respect to the prevention of slow flow. Thus, operators may extend the single session time up to 20–30 s if needed.
